# Phloem-mobile messenger RNAs and root development

**DOI:** 10.3389/fpls.2013.00257

**Published:** 2013-07-17

**Authors:** David J. Hannapel, Pooja Sharma, Tian Lin

**Affiliations:** Plant Biology Major, Iowa State UniversityAmes, IA, USA

**Keywords:** auxin, hormones, mobile RNAs, non-cell-autonomous, potato, StBEL5

## Abstract

Numerous signal molecules move through the phloem to regulate development, including proteins, secondary metabolites, small RNAs and full-length transcripts. Several full-length mRNAs have been identified that move long distances in a shootward or rootward direction through the plant vasculature to modulate both floral and vegetative processes of growth. Here we discuss two recently discovered examples of long-distance transport of full-length mRNAs into roots and the potential target genes and pathways for these mobile signals. In both cases, the mobile RNAs regulate root growth. Previously, RNA movement assays demonstrated that transcripts of *StBEL5*, a transcription factor from the three-amino-loop-extension superclass, move through the phloem to stolon tips to enhance tuber formation in potato (*Solanum tuberosum* L.). *StBEL5* mRNA originates in the leaf and its movement to stolons is induced by a short-day photoperiod. Movement of *StBEL5* RNA to roots correlated with increased growth and the accumulation of several transcripts associated with hormone metabolism, including *GA2-oxidase1, YUCCA1a* and *-c*, several *Aux/IAA* types, and *PIN1, -2*, and *-4* was observed. In another example, heterografting techniques were used to identify phloem-mobile *Aux/IAA* transcripts in *Arabidopsis*. Movement assays confirmed that these *Aux/IAA* transcripts are transported into the root system where they suppress lateral root formation. Phloem transport of both *StBEL5* and *Aux/IAA* RNAs are linked to hormone metabolism and both target auxin synthesis genes or auxin signaling processes. The mechanisms of transport for these mobile RNAs, the impact they have on controlling root growth, and a potential transcriptional connection between the BEL1/KNOX complex and *Aux/IAA* genes are discussed.

## Introduction

### Mobile signals to the root

The phloem functions as a remarkably efficient conduit for transferring numerous signal molecules throughout the plant. Proteins, metabolites, small RNAs and messenger RNAs move readily through the sieve element system to regulate development and respond to environmental changes. Some of the best examples include the Flowering Locus T protein that mediates flowering (reviewed by Turck et al., 2008) and GAI and Knotted1-like transcripts that regulate SAM development and leaf architecture (Kim et al., 2001; Haywood et al., 2005). These signal molecules function by moving through the phloem in a shootward direction but there are also good examples of shoot-to-root signaling as well. These basipetally directed signals have been implicated in controlling development and in responding to nutritive stress. Well-documented cases range from leaf-derived signals for nodule formation to microRNAs that communicate nutritional imbalances.

Nodule formation in legumes is tightly controlled by an intricate root-to-shoot-to-root signaling loop termed autoregulation of nodulation (Ferguson et al., 2010). This long-distance signaling pathway includes peptide hormones, receptor kinases and small metabolites. During nodule formation, a specific peptide hormone is transported from roots to leaves triggering the production of a leaf-derived signal that moves down into roots to suppress further nodulation. As another example, by visualizing radiolabeled hormones in young seedlings, cytokinins have been implicated as signals that move basipetally via symplastic connections in the phloem into roots (Bishopp et al., 2011). This long-distance basipetal transport of cytokinin is critical in regulating polar auxin transport and maintaining the vascular pattern in the root meristem. Phloem-derived miRNAs have also been established as information molecules with the capacity to move down into roots. During phosphate deprivation, miR399 moves from shoots to roots to enhance inorganic phosphate (Pi) uptake and translocation (Lin et al., 2008). This miRNA targets the ubiquitin-conjugating E2 enzyme 24, designated PHO2, suppressing its activity (Aung et al., 2006). PHO2 functions to repress Pi uptake and a *pho2* mutant over-accumulates Pi (Dong et al., 1998). Overall, the interaction between miR399 and PHO2 plays a crucial role of in the maintenance of Pi homeostasis. The phloem-mobile miR395 appears to operate in a similar fashion as a general component of the regulatory network of sulfate assimilation (Buhtz et al., 2010; Matthewman et al., 2012). Small RNAs involved in gene silencing also move from shoot to root across grafts and have the capacity to direct epigenetic modifications in the genome of recipient cells that can influence growth and development (Molnar et al., 2010; Bai et al., 2011; Melnyk et al., 2011).

### Functional phloem-mobile RNAs

Either by phloem cell microdissection or analysis of phloem sap, the transcriptome of phloem is revealed to include thousands of full-length mRNAs with a wide range of potential functions (Omid et al., 2007; Deeken et al., 2008; Kehr and Buhtz, 2008). Despite the fact that so many mRNAs have been identified in phloem sap, the movement of only a few has been confirmed to be associated with a function. These include *StBEL5* (Banerjee et al., 2006) and *POTH1* (Mahajan et al., 2012) of potato, *CmGAI* of pumpkin (Haywood et al., 2005), *PFP-LeT6* from tomato (Kim et al., 2001), and *AUX/IAA* (Notaguchi et al., 2012) and *FLOWERING LOCUS T* (Li et al., 2011; Lu et al., 2012) from *Arabidopsis*. Whereas, there are numerous examples of acropetal movement of mRNAs confirmed through grafting experiments (Kim et al., 2001; Haywood et al., 2005; Kanehira et al., 2010; Yang and Yu, 2010), there are only a few examples of full-length RNAs moving in a rootward direction. *GAI* transcripts of apple exhibited the capacity to move in both directions across a graft union, from scion to stock and from stock to scion (Xu et al., 2010). Two specific transcript types, *AUX/IAA* and *StBEL5*, have been shown to move into roots and affect a phenotype. Both are involved in auxin synthesis or signaling. The mechanisms of their movement and the potential regulatory networks they affect are the topics of this report.

### *AUX/IAA* mRNAs move into roots and suppress growth

AUX/IAA (Auxin/indoleacetic acid) proteins are important transcriptional regulators involved in auxin signaling (Tiwari et al., 2001). In general, they act as repressors by interacting with auxin response factors bound to auxin response elements of target genes that control numerous aspects of growth (Tiwari et al., 2001, 2004). Two types of *AUX/IAA* transcripts were first identified in phloem sap of melon (*Cucumis melo*). These melon RNAs were detected in the scion of heterografts of pumpkin/melon confirming that specific *AUX/IAA* RNAs were capable of long-distance trafficking (Omid et al., 2007). Using tobacco/*Arabidopsis* heterografts, a subsequent screen of the AUX/IAA family in *Arabidopsis* revealed two AUX/IAA RNAs, designated *IAA18* and −28, capable of long-distance transport (Notaguchi et al., 2012). Using a GUS marker in transgenic lines, promoter activity of both of these genes was localized to leaf vascular tissue.

To assess the function of phloem-mobile *IAA18* in root formation, the gain-of-function mutant, *diaa18*, was used in heterografts with WT stocks (Notaguchi et al., 2012). This dominant mutant, in which the protein is not targeted for degradation by the 26S proteasome, exhibits stable repressor activity in the auxin signaling cascade (Chapman and Estelle, 2009; Vanneste and Friml, 2009). In this model, because the IAA18 protein is not degraded in the *diaa18* mutant, it functions to repress root growth even in the presence of auxin. When IAA18 is degraded, auxin response factors are released and auxin activity and lateral root growth are enhanced (Notaguchi et al., 2012). Previous work showed that *diaa18* plants exhibited severe defects in lateral root formation, reflective of its role in root development (Rogg et al., 2001; Fukaki et al., 2002; Uehara et al., 2008; Péret et al., 2009; Notaguchi et al., 2012). Micrografting experiments showed that the capacity of *diaa18* to repress root growth was transmitted from the scion to the rootstock (Notaguchi et al., 2012). This repression of root growth by the *diaa18* scion occurred both with and without auxin treatments.

RNA movement assays utilizing heterografts of WT and double mutants demonstrated that transcripts of both *IAA18* and −28 moved from WT scions (Col-0) into both primary and lateral roots of stocks of the double mutant *iaa18;iaa28* (Notaguchi et al., 2012). These results clearly establish the long-distance transport of both *IAA* types and a strong correlation of *IAA18* movement with repression of root growth. Using a virus vector system and a Myc protein tag, Notaguchi et al. (2012) showed that, whereas *IAA18* and −28 transcripts could move through the phloem, neither of their respective proteins has the capacity to enter the sieve tube system. These results suggest that these two proteins are unlikely to function as long-distance signaling agents in *A. thaliana* and are consistent with the established instability of these proteins (Rogg and Bartel, 2001).

### *StBEL5* is transported into roots and affects growth

Previous work has clearly established the mRNA of StBEL5, a transcription factor of the three-amino-loop-extension superclass, as phloem mobile and enhancing the tuberization process in potato (*Solanum tuberosum* L.) by targeting genes that control growth (Banerjee et al., 2006). Working in tandem with the Knotted1-type transcription factor, POTH1, StBEL5 mediates vegetative development by regulating hormone levels (Chen et al., 2003). The BEL5/POTH1 complex binds specifically to a double TTGAC core motif present in target gene promoters (Chen et al., 2004).

RNA movement assays demonstrated that *StBEL5* transcripts move through the phloem to stolon tips, the site of tuber induction. *StBEL5* mRNA originates in the leaf, and its movement to stolons is induced by a short-day photoperiod. Recent work has established that *StBEL5* also moves into roots and affects root growth. Movement into underground organs has been assayed in several studies in a transgenic line, designated GAS:BEL5, that transcribes *StBEL5* in leaves only (Banerjee et al., 2006, 2009; Lin et al., 2013). Using the leaf-specific galactinol synthase (GAS) promoter (Ayre et al., 2003) to drive *StBEL5* expression and a transgenic RNA-specific sequence tag, movement of *StBEL5* RNA into other organs may be readily monitored by using qRT-PCR. In this system, any RNA driven by the GAS promoter and detected in organs other than the leaf is the result of long-distance transport. This promoter construct can essentially separate accumulation by transcription from accumulation by movement. These GAS:BEL5 lines are robust, slightly dwarf [probably due to the activation of GA2ox1 expression in GAS:BEL5 leaves (Lin et al., 2013)], and exhibit normal leaf morphology and enhanced root and tuber development. This phenotype was similar to 35S:BEL5 OE lines except for the shorter plant stature (Chen et al., 2003).

**Figure 1:**
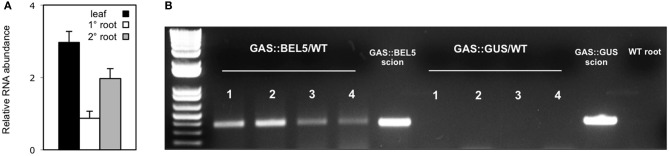
**Movement of transgenic *StBEL5* mRNA from leaf to root.** Quantification of movement was performed on transgenic lines expressing full-length *StBEL5* RNA driven by the galactinol synthase (GAS) promoter of melon (*Cucumis melo*) **(A)**. This promoter is predominately expressed in the minor veins of leaf mesophyll but not in other parts of the plant (Ayre et al., 2003; Banerjee et al., 2009). Relative levels of *StBEL5* RNA were quantified **(A)** from total RNA extracted from new leaves (■), and from either primary (□) or secondary (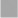) root samples of a SD-grown GAS:BEL5 transgenic plants. One-step RT-PCR was performed using 200–250 ng of total RNA, a primer for the NOS terminator sequence specific to all transgenic RNAs and a gene-specific primer for the full-length *StBEL5* transcript. These primers specifically amplify only transgenic *BEL5* RNA. All PCR reactions were standardized and optimized to yield product in the linear range. Homogenous PCR products were quantified by using ImageJ software (Abramoff et al., 2004) and normalized by using 18S rRNA values. Standard errors of the means of three replicate samples are shown. For heterografts **(B)**, micrografts were performed with replicates of either GAS:BEL5 scions on WT *andigena* stocks or GAS:GUS scions on WT *andigena* stocks. After 2 weeks in culture, grafts were moved to soil and grown under LDs for 3 weeks and then under SDs for 2 weeks before harvest of roots and leaves. After RNA extraction, RT-PCR with gene-specific primers was performed on RNA from WT lateral roots of both heterografts. A second PCR was performed with nested primers for both types. RNA from scion leaf samples was used as a positive control (scion samples). Two different gene-specific primers were used with a non-plant sequence tag specific for the transgenic *StBEL5* RNA to discriminate from the native RNA. Four plants were assayed for both heterografts and are designated 1–4. Wild-type RNA from lateral roots of whole plants (*S. tuberosum* ssp. *andigena*) was used in the RT-PCR reactions with *StBEL5* transgenic GSPs as a negative control (WT root). Similar negative results were obtained with RNA from WT leaves. (reprinted from Figure 1 of Lin et al., 2013; Copyright American Society of Plant Biologists, www.plantphysiol.org).

**Figure 2:**
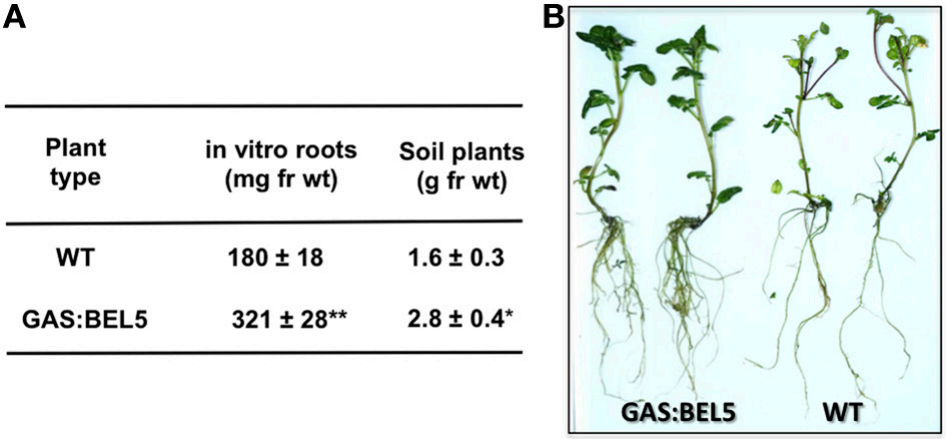
**Root development of transgenic lines of *Solanum tuberosum* ssp. *andigena* grown *in vitro* and in soil.** For root fresh weight harvests **(A)**, *in vitro* plantlets were grown for 4 weeks at 27°C under 16 h light, 8 h dark. Roots from *in vitro* transgenic lines were generally longer and more robust than WT controls **(B)**. Soil plants were grown in pots in a growth chamber under long days (16 h light, 8 h dark) at 24°C days and 18°C nights and harvested after 7 weeks. The SE of the mean of several plants is shown **(A)**. One asterisk indicates a significant difference (*p* < 0.05) and two asterisks, a significant difference (*p* < 0.01) using a Student's *t* test. (Reprinted from Figure 2 of Lin et al., 2013; Copyright American Society of Plant Biologists, www.plantphysiol.org).

Using both movement assays in whole plants (Figure 1A) and heterografts of GAS:BEL5 scions and WT stocks (Figure 1B), movement of transgenic *StBEL5* into roots was tested. The GAS promoter drives leaf-specific expression (Ayre et al., 2003) and in whole transgenic GAS:BEL5 plants, substantial amounts of transgenic *StBEL5* RNA were transported into both primary and lateral roots (Figure 1A). To confirm this movement, heterografts of GAS:BEL5 scions and WT stocks were performed and RT-PCR assays were used to detect the *StBEL5* transgenic RNA in the roots of WT stock material (Figure 1B). As a negative control GAS:GUS transgenic lines were grafted as scions onto WT stocks. Transgenic *StBEL5* RNA was detected in lateral roots of WT stock from four separate GAS:BEL5/WT heterografts whereas, no GUS RNA was detected in lateral roots from WT stock from four separate GAS:GUS/WT heterografts (Figure 1B). In correlation with the long-distance transport of transgenic *StBEL5* into roots, root growth was enhanced in the transgenic GAS:BEL5 lines in both soil-grown and *in vitro* plants by approximately 75% (Figure 2A). Root growth from these transgenic lines was more vigorous and robust than in WT lines (Figure 2B).

### Auto-regulation of *StBEL5* in roots

**Figure 3:**
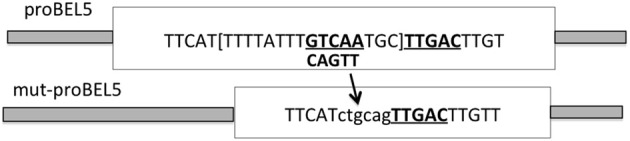
**Schematic of the modification of the wild-type *StBEL5* promoter sequence.** To create the mutated *BEL5* promoter used in the transgenic lines reported here, one of the tandem TTGAC *cis*-elements (underlined and bold) that make up the binding motif for StBEL5 and its Knotted1-like partner, POTH1 (Chen et al., 2004), was deleted. To facilitate cloning, this 5-base motif plus the TGC linker and eight other bp (all in brackets) were removed and replaced by the *ctgcag* sequence. The intact wild-type double motif sequence begins 820 nt upstream from the start of the *StBEL5* 5′ untranslated region (Chatterjee et al., 2007). (Reprinted from Figure 6 of Lin et al., 2013; Copyright American Society of Plant Biologists, www.plantphysiol.org).

Examination of the upstream sequence of the *StBEL5* promoter revealed the presence of the BEL/Knox tandem TTGAC motif (Chen et al., 2004) with the two core motifs on opposite strands of the DNA (Lin et al., 2013). This discovery suggested the possibility that the *StBEL5* gene could be auto-regulated. To test this possibility, two promoter constructs of *StBEL5*, both approximately 2.0 kb in length, were designed and fused to GUS, one with both motifs intact (proBEL5) and another with one of the motifs deleted, designated mut-proBEL5 (Figure 3). In transgenic lines driving GUS expression with the WT proBEL5 construct, activity was detected in both primary and lateral roots and in the phloem and cortex of primary roots (Lin et al., 2013). In the transgenic lines with the mut-proBEL5 construct, GUS activity was greatly reduced in lateral roots and in the phloem and cortex of primary roots. Overall, these results suggest that auto-regulation of the *StBEL5* gene is occurring in roots and that this expression is localized to cortical and phloem cells. Consistent with these observations, levels of endogenous *StBEL5* RNA are increased 2.4-fold in lateral roots of GAS:BEL5 plants relative to *StBEL5* in WT roots (Lin et al., 2013). Figure 1A shows the relative level of transgenic *StBEL5* RNA that is transported into these lateral roots.

## Results

### Long-distance transport of *StBEL5* into roots targets gene expression

Because of the role the StBEL5/KNOX complex plays in targeting genes involved in hormone synthesis and signaling pathways and with recent results confirming StBEL5′s effect on the induction of *GA2-oxidase1, YUCCA1a*, and *isopentenyl transferase* (*IPT*) in roots (Lin et al., 2013), a search for tandem TGAC motifs present in upstream sequence of several hormone genes was undertaken. Sequences meeting the criteria of a double motif present within 2.0 kb of the start codon (*LAX4* was the only exception) included three *PIN* genes (*PIN1*, −2, and −4), two *YUCCA1* genes (*1a* and *1c*), two *LIKE-AUX1* genes (*LAX1* and −4), one auxin response factor (*ARF8*), and *AGL8*, which is the potato MADS box gene, *POTM1-1*, shown previously by Rosin et al. (2003) to be involved in cyokinin regulation (Table 1). These elements contained linkers between the core motifs ranging from 2 to 24 nt. The motifs of *YUCCA1c* and *LAX4* contained no linker. All four 5′ to 3′ DNA strand orientations for the two core motifs are represented by this group: head-to-head, tail-to-tail, head-to-tail, and tail-to-head (Table 1).

**Table 1:** **Target genes of StBEL5 with tandem TTGAC core motifs in the upstream sequence**.

**Gene**	**Motif**	**No.**	**Orientation**	**Nt upstream**	**Comments**	**Promoter source**
StBEL5	**GTCAA**tgcTTGAC^*^	1	HtH	820 (TSS)	BEL5/POTH1 bind	Andigena
StPIN1	**TTGAC**actgagtttttcgatt**GTCAA TTGAC**ctacatacaatctGTCAA^*^	2	TtT TtT	1249 (AUG) 914 (AUG)	Auxin efflux	Phureja
StPIN2	**GTCA**ctatGTCAA^*^	1	HtT	1343 (AUG)	Auxin efflux	Phureja
StPIN4	**TGAC**actttca**GTCA**	1	TtT	486 (AUG)	Auxin efflux	Phureja
StGA2ox1	**TTGAC**aaGTCA^*^	2	TtT	1768 (AUG)	double, palindromic motif	Phureja
YUCCAla	**TTGAC**cttaTTGAC^*^	1	TtH	641 (AUG)	Auxin synthesis	Phureja
YUCCAlc	TGACTTGAC^*^	1	TtH	651 (AUG)	Auxin synthesis	Phureja
IPT	**TTGAC**aaGTCA^*^ GTCAAtgca**TGAC**	2	TtT HtH	1408(AUG) 568 (AUG)	OsKn1 OE lines induce IPT RNA	Phureja
LAX1	**TTGAC**ttttgatctTTGAC^*^	1	TtH	922 (AUG)	Auxin influx	Phureja
LAX4	**TTGACTGAC**	1	TtH	2629 (AUG)	Auxin influx	Phureja
ARF8	**GTCAA**ctccacaat**GTCA**	1	HtT	138 (AUG)	Auxin response factor	Phureja
AGL8	**GTCA**ttttcttcaatttgtctcgcttgt**GTCA**	1	HtT	1990 (TSS)	POTM1-1	Phureja

The TGAC core motifs running 5′ to 3′ are in bold letters. Linker sequence between the motifs is shown in lower case letters. The location of the motif is designated upstream from either the transcription (TSS) or the translation (AUG) start site. Orientation of the two motifs are designated: HtH, head-to-head; TtT, tail-to-tail; TtH, tail-to-head; or HtT, head-to-tail. An asterisk indicates binding to the BEL5/POTH1 complex via gel-shift assays was confirmed.

**Figure 4:**
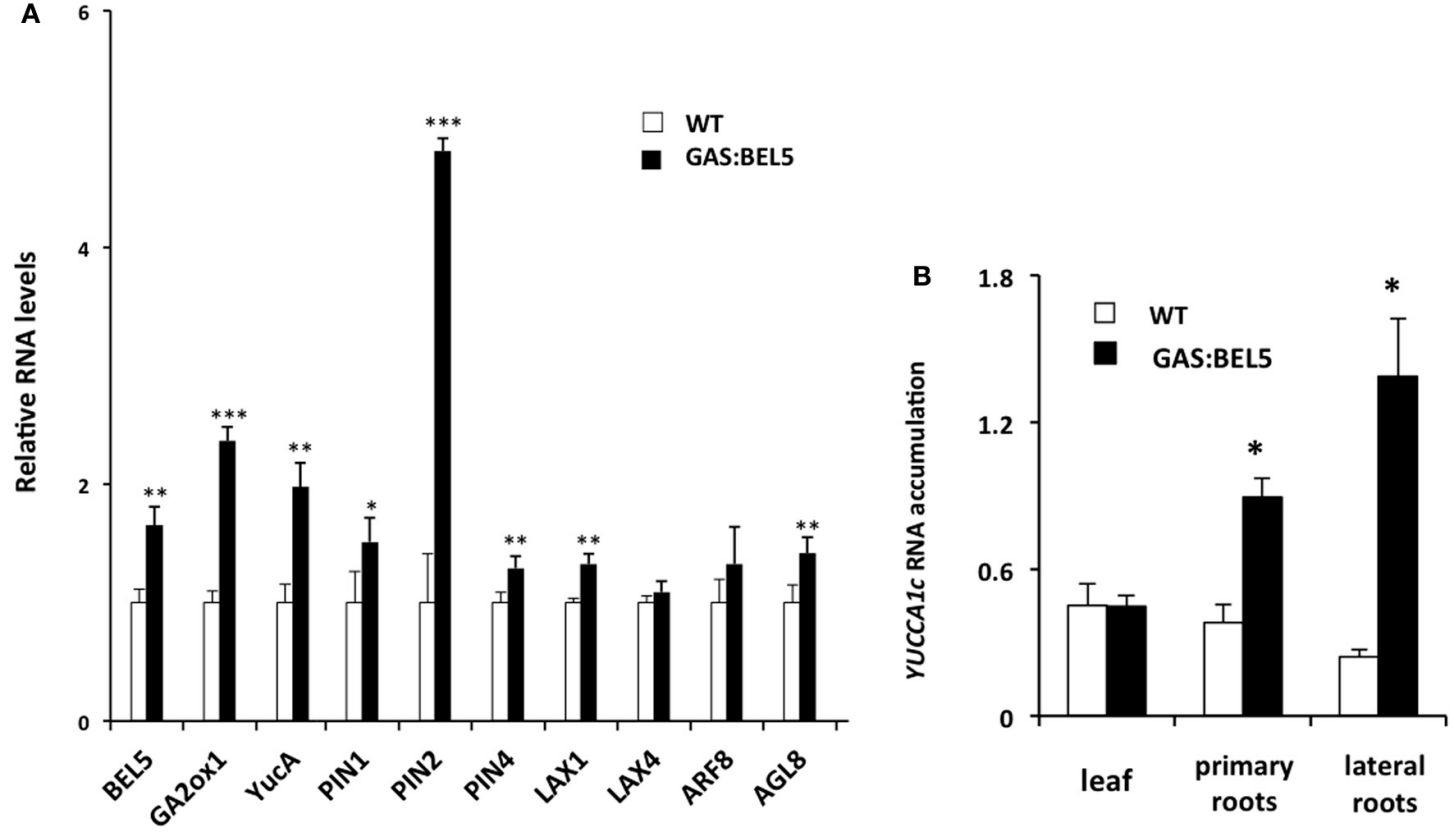
**RNA accumulation of several genes in secondary roots of GAS:BEL5 plants (A).** In these transgenic lines (■), transgenic *StBEL5* transcripts accumulate in roots (Lin et al., 2013). Quantitative real-time RT-PCR with gene-specific primers was used to calculate the relative amounts of each RNA **(A)**. *StBEL5* was included as a positive control. Each sample was measured in triplicate and normalized against *StActin8* RNA. The fold change in expression was calculated as the comparative threshold cycle method value relative to the mean values obtained from the WT samples (□). *YUCCA1c* induction in primary and lateral roots of GAS:BEL5 plants **(B)**. Quantification of mRNA in these samples was performed as described by Lin et al. (2013). Standard deviations of the means of three biological replicates are shown with one, two, and three asterisks indicating significant differences (*p* < 0.05, *p* < 0.01, *p* < 0.001, respectively) using a Student's *t*-test. YucA, YUCCA1a.

To assay RNA levels of target induction by mobile transgenic *StBEL5*, RNA samples were taken from lateral roots of the same soil-grown plants used in Figure 1A and compared to target RNAs from WT roots. In this phloem-transport induction system, GAS:BEL5 transgenic lines express *StBEL5* only in leaves so that any *BEL5* RNA detected elsewhere (e.g., roots) represents long-distance transport. qRT-PCR was performed on eight of the potato genes of Table 1 plus *StBEL5, YUCCA1a*, and *GA2-oxidase1* as controls. StBEL5 induces expression of *YUCCA1a, GA2oxidase1* and its own gene in lateral roots (Lin et al., 2013). Six of the candidate genes exhibited induction in response to *StBEL5* accumulation (Figures 4A,B). Levels of *LAX4* and *ARF8* exhibited no significant increase. Both *PIN2* and *YUCCA1c* transcript levels increased by approximately 5-fold.

**Figure 5:**
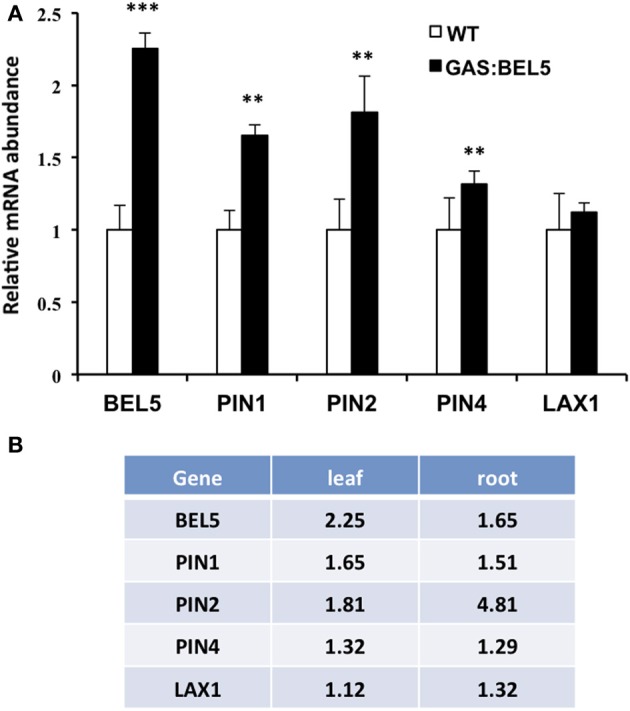
**Accumulation of RNA for select target genes in leaves of GAS:BEL5 plants (A) and a comparison of values in both leaves and roots (B).** In these transgenic lines (■), transgenic *StBEL5* transcripts are transcribed in leaves and move to roots (Lin et al., 2013). Quantitative real-time RT-PCR with gene-specific primers was used to calculate the relative amounts of each RNA. *StBEL5* was included as a positive control. Each sample was measured in triplicate and normalized against *StActin8* RNA. The fold change in expression was calculated as the comparative threshold cycle method value relative to the mean values obtained from the WT samples (□). The WT value is equivalent to 1.0. Standard deviations of the means of three biological replicates are shown with two and three asterisks indicating significant differences (*p* < 0.01 and *p* < 0.001, respectively) using a Student's *t*-test.

Because the GAS:BEL5 line expresses *StBEL5* in the leaves, the possibility exists that these target RNAs are up-regulated in leaves and may either move down to roots or activate pathways that lead to their induction in roots. Previous work with this system showed that several genes activated by *StBEL5* in roots or stolons were not induced in leaves. These included *YUCCA1a* and *-c, ISOPENTENYL TRANSFERASE*, and *StBEL5* and −22 (Lin et al., 2013, Figure 4B). One notable exception is *GA2ox1* which is induced in both leaves and roots (Lin et al., 2013). As previously discussed, this increase in leaves may very likely explain the slight dwarf phenotype exhibited by GAS:BEL5 transgenic lines. An assay for leaf RNA of the four induced auxin genes, *StPIN1*, −2, −4, and *StLAX1*, in the same GAS:BEL5 line used for root induction was performed (Figure 5A). There was no induction of *StLAX1* in leaves but the three *PIN* genes showed slight increases of their transcript levels in leaves that corresponded very closely to their induction levels in roots (Figure 5B). The one exception was *StPIN2* which exhibited a 1.8-fold increase in leaves but a 4.8-fold increase in roots. This very high level of root-specific accumulation was also reported for the tomato ortholog of *PIN2* (Pattison and Catala, 2012). The correlation in transcript levels in both leaves and roots for *StPIN1* and −4 would suggest a similar transcriptional relationship. It is conceivable that *StPIN2* transcripts are transported to roots but to date there is no report of any phloem-mobile *PIN* mRNAs.

**Figure 6:**
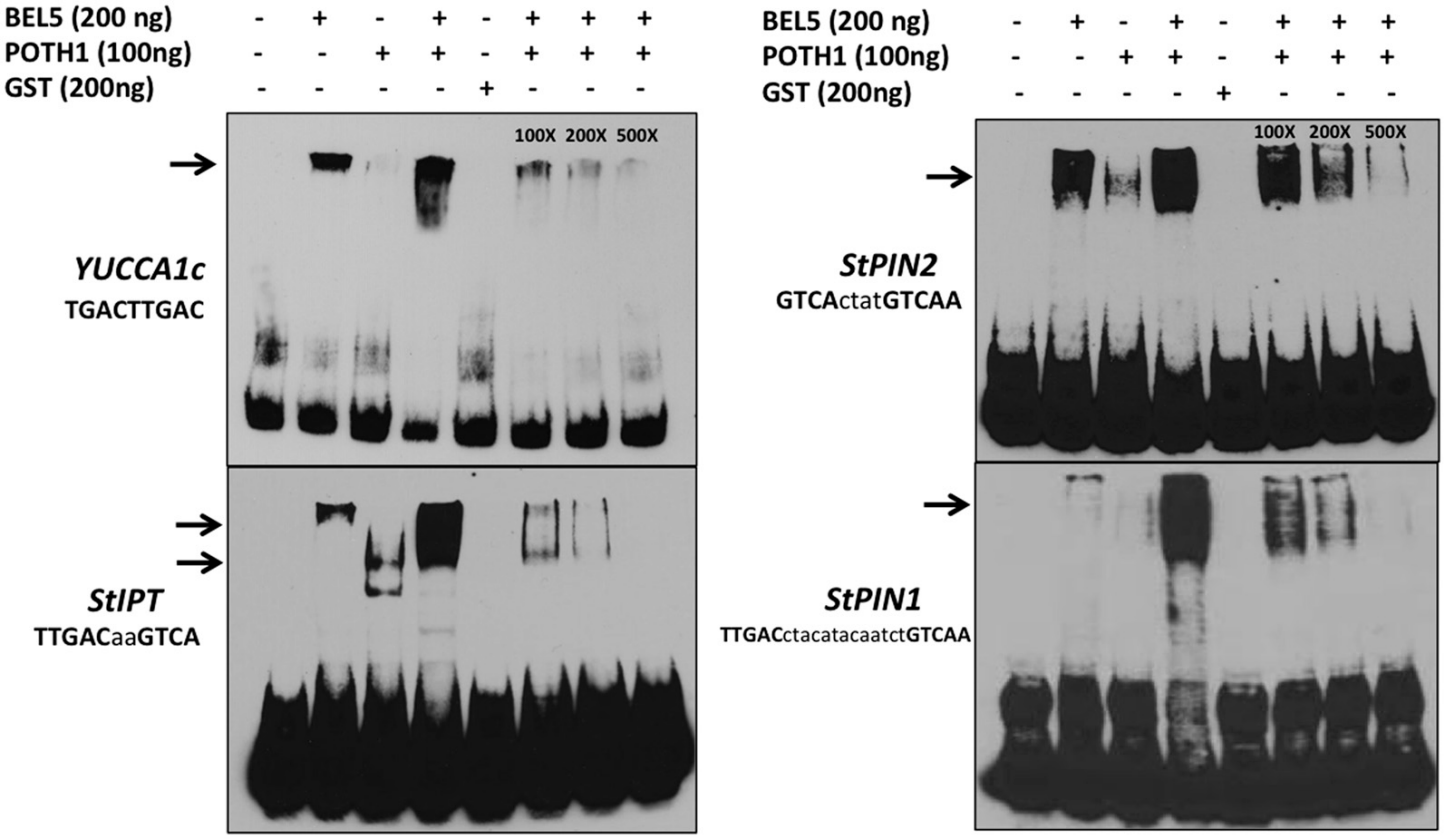
**Gel-shift assays of various tandem TGAC core motifs (bold, upper case nucleotides) in four putative target genes of StBEL5 and POTH1 with a range of linker sequence (lower case nucleotides) between motifs.** Included here are *YUCCA1c, StIPT* (*isopentenyl transferase*), and *StPIN1* and −2. The StBEL5 and POTH1 proteins were expressed and purified with a carboxyl-terminal glutatione S-transferase (GST) fusion tag. Each DNA bait was tested for binding with StBEL5-GST, POTH1-GST or GST alone or with StBEL5-GST and POTH1-GST together. Ten fm of synthesized DNA probes 50 nt in length labeled with biotin were used in the binding reaction. The amounts of StBEL5 and POTH1 proteins used in these assays were adjusted to achieve equivalent molarity. Unlabeled DNA bait at 100X, 200X, and 500X concentrations relative to the labeled probe was used in the competition assays. Arrows indicate approximate location of shifted bands.

To determine if the StBEL5/POTH1 complex interacts with the double elements identified in upstream sequence of the induced genes, gel-shift assays were undertaken on select targets from Table 1 and Figure 4 (Figure 6). The four *cis*-elements tested for binding represented three of the four strand orientations: tail-to-head for *YUCCA1c*, tail-to-tail for *StIPT* and *StPIN1*, and head-to-tail for *StPIN2*. The linker region between the TTGAC core motifs of these four elements ranges from no linkers for *YUCCA1c* to a 13-nt linker for the *StPIN1* motif. *StIPT* and *StPIN2* contain linkers of 2 and 4 nt, respectively. Despite the diversity in strand orientation and linker length, binding of proteins to these elements was consistently strongest for the BEL5/POTH1 complex and the StBEL5 protein alone (Figure 6). No interaction was observed with the glutatione S-transferase (GST) protein alone.

### Induction of *AUX/IAA* in roots of GAS:BEL5 plants

**Figure 7:**
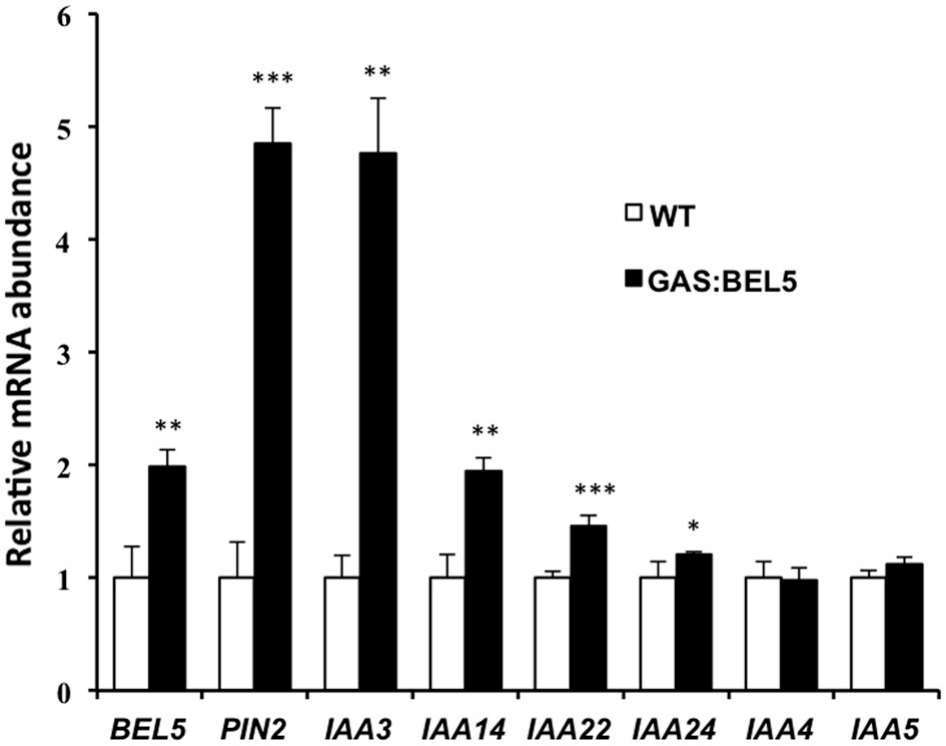
**RNA accumulation of several *StAUX/IAA* genes in secondary roots of GAS:BEL5 plants.** In these transgenic lines (■), transgenic *StBEL5* transcripts accumulate in roots (Lin et al., 2013). Quantitative real-time RT-PCR with gene-specific primers was used to calculate the relative amounts of each RNA. Each sample was measured in triplicate and normalized against *StActin8* RNA. The fold change in expression was calculated as the comparative threshold cycle method value relative to the mean values obtained from the WT samples (□). Standard deviations of the means of three biological replicates are shown with one, two, and three asterisks indicating significant differences (*p* < 0.05, *p* < 0.01, *p* < 0.001, respectively) using a Student's *t*-test. *StBEL5* and *StPIN2* were included as positive controls. *StIAA4 and -5* do not contain a double TTGAC motif in their upstream sequence (Table 2).

Using ChIP-seq, targets of KNOTTED1 in maize included many genes involved with gibberellic acid, brassinosteroid, cytokinin, and auxin synthesis and signaling pathways (Bolduc et al., 2012). Among the target hormonal genes that bound to KNOTTED1, differential gene expression occurred preferentially for auxin-related genes, including transcription factors involved in auxin signaling like *AUX-IAA* and *ARFs*. KNOTTED1 binds to eighteen *AUX/IAA* and twenty *ARF* genes, almost half of the *AUX-IAA* and *ARF* genes identified in the maize genome (Bolduc et al., 2012; Supplemental Table 7). The ChIP-seq analysis further demonstrated that KNOX transcription factors have degenerate binding sites and that specificity may be acquired through an interaction with binding partners like the BEL1-type transcription factors. In potato, the BEL1/KNOX interaction has been well-documented (Chen et al., 2004; Lin et al., 2013). Of the twenty-seven potato *AUX/IAA* genes, fourteen contain the tandem TGAC core motif representing all four orientations in their upstream sequences (Table 2). These observations coupled with the results of Bolduc et al. (2012) suggest the intriguing possibility that a connection exists between the BEL1/KNOX transcriptional complex and *AUX/IAA* gene activity. To test this hypothesis, induction of select *StAUX/IAA* genes was assayed in the roots of the GAS:BEL5 line. Four *StIAA* genes, *IAA3*, −14, −22, and −24, that contained the double TTGAC motif (Table 2) also exhibited induction in correlation with an increase in *StBEL5* transcripts, whereas two genes without double TTGAC motifs in their upstream sequence, *StIAA4* and −5, exhibited no such increase (Figure 7). The group of genes showing positive induction represented all four motif orientations (tail-to-head, head-to-tail, head-to-head, and tail-to-tail, respectively) with linker sequence between TGAC cores ranging from 0 (*StIAA3*) to 12 (*StIAA24*) nt. Induction levels of the four *StIAA* genes exhibited a negative correlation with the length of the linker sequence suggesting that linker length may influence rate of transcriptional activity. The strongest induction was with *StIAA3* and levels of induction decreased steadily as the linker length increased from 0 to 1, 4, and 12 nt (Figure 7, Table 2).

**Table 2:** **Tandem TGAC core motifs present in the upstream sequences of *AUX/IAA* genes of potato**.

**Potato IAA # (Wu et al., 2012)**	**Arabidopsis ortholog**	**Potato gene ID**	**TTGAC element**	**Orientation**	**Base pairs from AUG**
1	IAA4	PGSC0003DMG400016317	**GTCAA**ctt**GTCA**	HtT	1546
2	IAA1	PGSC0003DMG400020139	no	—	—
3	IAA1	PGSC0003DMT400049677	**TTGACTTGAC**	TtH	1394
4	IAA9	PGSC0003DMG400006393	no	—	—
5	IAA13	PGSC0003DMG400029339	no	—	—
6	IAA8	PGSC0003DMG400002550	**TGAC**ctaat**TTGAC**	TtH	2524
7	IAA17	PGSC0003DMG400016280	no	—	—
8	IAA16	PGSC0003DMG402002635	no^*^	—	—
9	IAA16	PGSC0003DMG402019457	**TGAC**ttattgc**TTGAC**	TtH	1674
10	IAA16	PGSC0003DMG400005327	no	—	—
11	IAA19	PGSC0003DMG400002636	no	—	—
12	IAA3	PGSC0003DMG400013445	**TTGAC**ataacaa**TTGAC**	TtH	794
13	ARF9	PGSC0003DMG400005794	no	—	—
14	IAA18/28	PGSC0003DMG400002608	**GTCA**t**GTCAA**	HtT	2091
15	ARF9	PGSC0003DMG400000118	**TGAC**tctaagacat**TTGAC**	TtH	1873
16	IAA19	PGSC0003DMG400016512	**GTCA**c**TTGAC**	HtH	2233
17	IAA3	PGSC0003DMG400005338	**GTCA**tttagatt**TTGAC**	HtH	1152
18	IAA29	PGSC0003DMG400020478	**TTGAC**acatttga**TGAC**	TtH	992
19	IAA29	PGSC0003DMG400030896	no	—	—
20	IAA33	PGSC0003DMG400043142	no	—	—
21	IAA29	PGSC0003DMG400013765	no	—	—
22	IAA10	PGSC0003DMG400008586	**GTCAA**ttaa**TTGAC**	HtH	712
23	IAA4	PGSC0003DMG400006108	no^*^	—	—
24	IAA14	PGSC0003DMG400006093	**TGAC**aatacataagaa**GTCAA**	TtT	667
25	IAA12	PGSC0003DMG400001498	**TTGAC**att**TTGAC**	TtH	2319
26	IAA27	PGSC0003DMG400000375	no	—	—
27	ARF16	PGSC0003DMG400021560	**GTCAA**-20 nt-**GTCAA**	HtT	2235
	StBEL5	PGSC0003DMG400005930	**GTCAA**tgc**TTGAC**	HtH	970

These twenty-seven genes were identified by Wu et al. (2012) and were designated numbers from 1 to 27 (first column). Arabidopsis orthologs are shown in the second column. The PGSC ID numbers are from the potato genome database. The TGAC core motifs running 5′ to 3′ are in bold letters. Linker sequence between the motifs is shown in lower case letters. The location of the motif is designated upstream from the translation (AUG) start site. Orientation of the two motifs are designated: HtH, head-to-head; TtT, tail-to-tail; TtH, tail-to-head; or HtT, head-to-tail. An asterisk indicates some upstream sequence was not available from the potato genome. The motif of StBEL5 is shown as a control. StBEL5 auto-regulates its own gene (Lin et al., 2013).

## Discussion

### IAA18 and StBEL5 regulate transcription

Two unique full-length messenger RNAs that originate in leaf vascular tissue, transverse graft unions to move into roots and impact growth. Both are transcription regulators. IAA18, a putative transcription repressor, dimerizes with auxin response factors (ARFs) to suppress their effect in enhancing auxin activity. In *Arabidopsis*, there are twenty-nine AUX/IAA genes and in rice and maize there are thirty-one (Liscum and Reed, 2002; Jain et al., 2006; Wang et al., 2010). Most AUX/IAA proteins contain four highly conserved domains that facilitate dimerization to target proteins and degradation of the AUX/IAA proteins via the ubiquitin-proteasome pathway (Dharmasiri et al., 2005). Under low auxin concentration, ARFs are inhibited by their interaction with domains III and IV of the AUX/IAAs (Ulmasov et al., 1997; Hagen and Guilfoyle, 2002). Elevated auxin levels releases ARFs from the repressor heterodimer by promoting the degradation of AUX/IAA proteins (Tiwari et al., 2001; Berleth et al., 2004; Dharmasiri et al., 2005). AUX/IAA proteins function in regulating lateral root formation, apical dominance, apical hook curvature, petiole epinasty, leaf architecture, and fruit development. In the model presented by Notaguchi et al. (2012), shoot-derived *IAA* transcripts are transported into the root tip and contribute to the regulation of lateral root formation. They propose that this regulation is accomplished by an interaction between IAA18 and cytokinin as negative repressors, and auxin as the positive regulator. Their results, however, do not rule out the possible involvement of other signaling agents, including other mobile *AUX/IAA* transcripts.

Whereas IAA18 functions as a transcription regulator through its interaction with ARFs, the mobile RNA, *StBEL5*, encodes a transcription factor that acts directly in tandem with a KNOTTED1-like partner to bind to conserved motifs in upstream sequence of numerous target genes that impact hormone synthesis and transport. StBEL5 is regulated in several ways. Its transcription is activated by low levels of white light and short days then facilitate long-distance transport of its mRNA (Banerjee et al., 2006; Chatterjee et al., 2007). RNA chaperone proteins bind to *StBEL5* RNA to potentially mediate movement, stabilize the RNA, and repress translation (Cho et al., 2012). Established as a chaperone for mobile RNAs in pumpkin, a polypyrimidine tract-binding (PTB) protein of potato binds to the 3′ untranslated region of *StBEL5* (Mahajan et al., 2012). Interaction with a PTB protein can mediate movement and repress translation until it is activated at functional sites in specific cells of roots and stolons. Similar to other BEL1-like transcription factors, StBEL5 is functional with an interactive KNOX partner that is co-expressed and co-localized (Bhatt et al., 2004; Cole et al., 2006).

### A network of controls

In a remarkable feedback process, auxin moves through the phloem into roots (Chhun et al., 2007) and controls degradation of AUX/IAA proteins, proteins encoded by phloem-mobile transcripts (Notaguchi et al., 2012). Now we add a third component, mobile transcripts of StBEL5, a transcription factor that targets and induces both biosynthetic (*YUCCA1*) and transport (*PIN, LAX*) genes in the IAA pathway. These layers of regulation suggest a complex and finely tuned network of control that has very likely evolved across species with fundamental conserved components. There are twenty-seven non-redundant *AUX/IAA* genes identified in potato (Wu et al., 2012; Table 2) but as yet, none have been confirmed as phloem-mobile. If any potato *AUX/IAA* transcripts are mobile, then one would expect their levels in phloem cells to be relatively high. Both *IAA18* and *StBEL5* are transcribed in phloem cells and subsequently loaded into sieve elements (Banerjee et al., 2006; Notaguchi et al., 2012). Based on RNA-Seq data, six of the twenty-seven *AUX/IAA* RNAs of potato, including the ortholog of *IAA18*, exhibit very high abundance levels in phloem cells comparable to two known mobile RNAs (Table 3). This data is consistent with the possibility that select *AUX/IAA* RNAs of potato may also be phloem mobile.

**Table 3:** **Abundance of *AUX/IAA* transcripts of potato from phloem-associated cells of petioles and stems**.

**Phloem source**	***IAA2***	***IAA3***	***IAA4***	***IAA6***	***IAA9***	***IAA10***	***IAA13***	***IAA14***	***IAA15***	***IAA21***	***IAA26***	***BEL5***	***GAI***
Petiole	49	0	7410	18	2762	3493	0	3571	10656	176	3262	9592	2545
Stem	106	0	13694	52	2531	2556	26	5245	11580	242	5116	5531	2092

The potato IAA genes were identified and numbered by Wu et al. (2012). Total counts were derived from RNA-Seq data from RNA extracted from laser-capture microdissected phloem-associated cells of petioles or stems from Solanum tuberosum ssp. andigena plants grown under a short-day photoperiod. Total count values represent the sum of three biological replicates. StBEL5 and StGAI are included as mobile RNA controls. Six StIAA RNAs exhibited abundance values for both petiole and stem phloem greater than the StGAI values. IAA14 is the AtIAA18/28 ortholog in potato.

Movement and localization of signals and elicitors are key issues when considering auxin's role in meristem development, vascular tissue development and the genesis of lateral roots. To add one more layer of control, StBEL5 auto-regulates its own expression in roots and stolons (Lin et al., 2013). StBEL5 transcription is localized to root phloem and accumulation of its mobile, transgenic RNA in roots was correlated with increased stele diameter of primary roots, anomalies in the organization of the vascular core, and increased lateral root formation (Lin et al., 2013). All three of these observations can be explained by changes in hormone activity of cytokinin, gibberellic acid, and auxin (Dettmer et al., 2009; Gou et al., 2010; De Smet, 2012). Induction assays showed that StBEL5 targets genes are involved in the metabolism of all three (Figures 4 and 7; Lin et al., 2013). Both zeatin and isopentenyl types of cytokinins increased in over-expression lines of StBEL5 in potato (Chen et al., 2003) and over-expression of BEL1-like genes produced plants that were dwarf due to decreases in active gibberellin levels (Dong et al., 2000; Müller et al., 2001). In similar fashion, a BEL1-like transcription factor was required for regulating both cytokinin and auxin signaling pathways, including PIN1 activity, to establish the correct pattern of ovule development in *Arabidopsis* (Bencivenga et al., 2012).

### A transcriptional connection?

ChIP-seq analysis revealed numerous target genes of KNOTTED1 in maize involved with hormone synthesis and signaling and demonstrated that KN1 plays a key role in a regulatory network that influences myriad aspects of development (Bolduc et al., 2012). The maize *ga2ox1*-like binding motif with two TGAC core motifs (Bolduc and Hake, 2009) was identified as an important element present within the KN1-bound regions. Several *AUX-IAA* and *ARF* genes were identified as targets as well. Because of the strong interaction of the StBEL5/POTH1 complex with TGAC core elements (Figure 6, Chen et al., 2004), it is plausible that BEL1-like transcription factors are also involved in many of the interactions documented in this ChIP-seq analysis. For example, at least three genes identified in the KNOTTED1 screen, *GA2ox1, IPT*, and *PIN1*, contain elements that bind to StBEL5/POTH1 and are induced by StBEL5 (Figures 4 and 6, Lin et al., 2013).

Although the strongest relative *in vitro* binding was observed with KNOTTED1 protein alone with tandem TGAC core motifs on the same strand of DNA with a 3-bp linker gap (Bolduc et al., 2012, Supplemental Table 4), we have consistently observed that the most robust gel shifts involved the POTH1/BEL5 heterodimer with motifs on both the same and opposite strands and with linkers between core motifs ranging from zero to 13 bp (Table 1; Figure 6; Chen et al., 2004; Lin et al., 2013). This discrepancy in binding affinity in response to the strand orientation of tandem motifs or linker length between motifs may be a function of the spatial dynamics of the KNOX/KNOX or POTH1/BEL5 dimers. Even though the homeodomains of KNOTTED1- and BEL1-like transcription factors are almost identical, molecular weight differences are significant. StBEL5 has a molecular weight of 76 kDa, whereas the KNOTTED1-types (including POTH1) are approximately 37–40 kDa. A BEL1/KNOX transcriptional complex may very well impart a degree of specificity and spatial flexibility not provided by a KNOTTED1 homodimer.

In summary, these results confirm that *AUX/IAA* RNAs like *IAA18* and *StBEL5* and its transcriptional partners are involved in a complex developmental network that regulates hormone activity in roots through the long-distance transport of their mRNAs. Despite the apparent complexity of this system, however, it is almost certain that other long-distance signaling agents, including phloem-mobile RNAs, are also functional in regulating root morphology and are awaiting discovery.

## Materials and methods

### Plant material

Soil-grown plants were maintained in a growth chamber under either a long-day (16 h light at 22°C, 8 h dark at 18°C) or a short-day (8 h light at 22°C, 16 h dark at 18°C for 12 days) photoperiod with a fluence rate of 400 μmol m^−2^ s^−1^. Leaves and roots from GAS:BEL5 plants were harvested from soil-grown plants at the 12- to 13-leaf stage, frozen in liquid nitrogen and stored at -80°C prior to RNA extraction. Primary and secondary root types could be distinguished by their morphology. Primary roots are thicker and often exhibit a light purple color, whereas secondary roots are bright white.

### Real time qRT-PCR

Total RNA was extracted from all the plant tissues using the RNeasy Plant Mini Kit (Qiagen, USA) according to manufacturer's instructions. RNA samples with 260/280 ratio from 1.9 to 2.1 and 260/230 ratio from 2.0 to 2.5 were used for qRT-PCR analysis. qRT-PCR analysis was performed with qScript™ One-Step SYBR Green qRT-PCR Kit (Quanta Biosciences) following manufacturer's protocol. Briefly, 50 ng aliquots of total RNA template was subjected to each qRT-PCR reaction in a final volume of 15 μl containing 7.5 μl One-step SYBR Green Master Mix and 0.3 μl of qScript One-step Reverse Transcriptase along with target specific primers (200 nM). All reactions were performed in triplicate using Illumina Eco qPCR machine (Illumina, USA) with fast qPCR cycling parameters (cDNA synthesis: 50°C, 5 min; Taq activation: 95°C, 2 min; PCR cycling (40 cycles): 95°C, 3 s/60°C, 30 s). *StACT8* (accession number GQ339765) was used as an endogenous control for normalization of the total RNA template in a reaction. The relative gene quantification (comparative threshold cycle) method (Livak and Schmittgen, 2001) was used to calculate the expression levels of different target genes. Primers ranged from 98 to 160 bp and were mostly designed spanning the introns in order to detect any genomic DNA contamination. Specificity of primers was determined by melting curve analyses and agarose gel (3%) electrophoresis performed following the qRT-PCR experiments. A standard curve was generated based on 6-point (10-fold) serial dilutions of cDNA to calculate the gene specific PCR efficiency. PCR efficiencies of primers ranged from 97 to 110%.

### Protein expression and purification

Glutathione S-transferase (GST) fusion constructs of StBEL5 and POTH1 described by Chen et al. (2004) were used for preparation of GST-tagged StBEL5 and POTH1. For GST-POTH1 expression, pGEX-POTH1 was transformed into BL21 Star (DE3) *E. coli* cells (Invitrogen, CA). Cells were grown at 37°C until the OD_600_ reached 0.6, induced with 1.0 mM IPTG and cultured at 30°C for 5 h. For expression of GST-StBEL5, pGEX-StBEL5 was transformed into ArticExpress *E. coli* cells (Agilent, CA). Cells were grown at 37°C until the OD_600_ reached 0.6, induced with 1.0 mM IPTG and cultured at 4°C for 48 h. The GST-tagged protein purification was performed using the Pierce GST Spin Purification kit.

### Gel-shift assays

Oligos with 3′ biotin labeling were synthesized by the DNA Sequencing and Synthesis Facility, Iowa State University, Ames, IA. Double-stranded DNA was prepared by hybridization of complementary synthetic oligonucleotides. Gel-shift assays were performed using the LightShift Chemiluminescent EMSA Kit from Thermo Scientific according to the manufacturer's protocol provided in the kit with the following modifications. Twenty ul DNA-binding reactions were set up on ice containing 20 mM HEPES (pH 7.5), 10% glicerol (V/V), 0.5% Triton X-100 (V/V), 0.5 mM EDTA (pH 8.0), 50 mM KCl, 2 mM MgCl_2_, 20 ng/μl BSA, 1 mM DTT, 50 ng/ul of poly(dI-dC) as a non-specific competitor. Ten fmol of labeled DNA was used for all targets. 200 ng of StBEL5, 100 ng of POTH1 and 200 ng of GST proteins were used as indicated in Figure 6. The binding system was incubated on ice for 60 min before electrophoresis. For the competitive assay, unlabeled double stranded DNA fragments were incubated with the recombinant protein on ice for 30 min before addition of the labeled probe.

### RNA-Seq analysis

RNA-Seq was performed on RNA extracted from laser-capture microdissected (LCM) phloem cells of potato (*S. tuberosum* ssp. *andigena*) plants grown under short-day conditions (Yu et al., 2007). RNA was isolated using the PicoPure™ RNA Isolation kit (Arcturus). RNA amplification using 70–260 pg of LCM RNA samples from three biological replicates from both stem and petiole sections was performed. The Ovation RNA-Seq kit (NuGEN) was used for cDNA synthesis. One ug of cDNA from each replicate sample was subjected to 100-cycle paired- or single-end sequence cluster generation on an Illumina HiSeq 2000 instrument at the DNA Facility, Iowa State University. The reads from sequencing were saved in fastq format and aligned to the potato genome (PGSC_DM_v3.4_gene.fasta & PGSC_DM_v3.4_gene.gff from http://solanaceae.plantbiology.msu.edu/pgsc_download.shtml) with Genomic Short-read Nucleotide Alignment Program (GSNAP). False Discovery rate (FDR) was set at 0.05. Read numbers were recorded for each replicate and analyzed for abundance. The longest peptide sequence was selected from the genome database for all the genes in both the stem and petiole lists (PGSC_DM_v3.4_pep_representative.fasta). Functions of these genes were analyzed with Blast2Go (http://www.blast2go.com/b2glaunch/start-blast2go).

### Primers and oligos used for qRT-PCR and gel-shift assays

#### Primers used for qRT-PCR

BEL5F: 5′-CTGCAACAGCTAGGAATGATG-3′

BEL5R: 5′-ATGATTTTGTCTGAATCCTTTGGG-3′

GA2ox1F: 5′- AGGCACAGAGTGATCGCAGAT-3′

GA2ox1R: 5′- TGGTGGCCCTCCAAAGTAAA-3′

YUCCA1aF: 5′-CATTATCACAAATAAAAACCGGAAA-3′

YUCCA1aR: 5′-TGCCATCTAAAAATCTTGCACC-3′

PIN1F: 5′- GCACCAAATCCTGGCATGT-3′

PIN1R: 5′- AGCTGTATTCTTGTGTGCTTTGGT-3′

PIN2F: 5′-GCAAGTTTGATTGGACTCATTTG-3′

PIN2R: 5′-TGAGATCGAACCTTTTACAATCG-3′

PIN4F: 5′- GTTTCATTGCGGCGGATTC-3′

PIN4R: 5′- CCCATAGCGAAAGAACAACC-3′

LAX1F: 5′- GCGCCTATCCACCACTAAGAAG-3′

LAX1R: 5′- GAAGTATTGCAACAACCCCATGTA-3′

LAX4F: 5′- GCCACCGTGCACAACTAGG-3′

LAX4R: 5′- CACTAACACATTTTGGGTTACATGC-3′

ARF8F: 5′- CAGCCTAAGCGGCATCTTCT-3′

ARF8R: 5′- AGCCTTTTGGCGCTAACAAA-3′

AGL8F: 5′- AGCAAAACAACCAGCTTTCCAA-3′

AGL8R: 5′- TGATCCCACTGATTTTGCTGTG-3′

YUCCA1cF: 5′-ACAAATACAAAGAGGTGTGTATTCGT-3′

YUCCA1cR: 5′-CGTAGTCATTGTATCCGTCCTGA-3′

ACTF: 5′-TGATTGGTATGGAAGCTGCAG-3′

ACTR: 5′-CCACTGAGCACAATGTTACCG-3′

StIAA3 Fw: CTGATCTTCGATCAATTTCATGG

StIAA3 Rv: GACCTATTGCTGCCTTGTGCTA

StIAA4 Fw: CCAGCATTACTATTAGGCGAGG

StIAA4 Rv: CCATGTCGTAATCAGGTAAAGC

StIAA5 Fw: GACTACTGAGGCCAAAGGACTTG

StIAA5 Rv: TGTGGTCTCATTTGATCATTTGC

StIAA14 Fw: TGATGTGAATGAGCTAACGAGATG

StIAA14 Rv: GCTTCTGCAACTACACTTGAACAA

StIAA22 Fw: TAGGTCAGACAAAGAATCAACTT

StIAA22 Rv: CCATCCACTAAAATTTCCTTCTA

StIAA24 Fw: AAGCCAATTGATGGTGTGCA

StIAA24 Rv: ATACAAGCACATGAAAACAACAA

#### Sequences of oligos used for gel shifts

YUCCA1cS: 5′-AAAAAATTACAAATAAATGACTTGACTAATGTTGTTATTAATCTCCACA-3′

YUCCA1cA: 5′-TGTGGAGATTAATAACAACATTAGTCAAGTCATTTATTTGTAATTTTTT-3′

IPTS: 5′-TTTTTTTTTGGTTTTAAGTTTGACAAGTCAGGTCTAATTTGACATCCTT-3′

IPTA: 5′-AGGATGTCAAATTAGACCTGACTTGTCAAACTTAAAACCAAAAAAAAA-3′

PIN1Fw: 5′-GTCTGTGTATGATTTTGACCTACATACAATCTGTCAACTAATGTGTATGA-3′

PIN1Rv: 5′-TCATACACATTAGTTGACAGATTGTATGTAGGTCAAAATCATACACAGAC-3′

PIN2Fw: 5′-AAATGTGAAAGTCACTATGTCAATCATTATTT-3′

PIN2Rv: 5′-AAATAATGATTGACATAGTGACTTTCACATTT-3′

### Conflict of interest statement

The authors declare that the research was conducted in the absence of any commercial or financial relationships that could be construed as a potential conflict of interest.
